# Expression of drug transporters in human kidney: impact of sex, age, and ethnicity

**DOI:** 10.1186/s13293-015-0020-3

**Published:** 2015-03-02

**Authors:** Stancy Joseph, Tamara J Nicolson, George Hammons, Beverly Word, Bridgett Green-Knox, Beverly Lyn-Cook

**Affiliations:** Division of Biochemical Toxicology, National Center for Toxicological Research, Food and Drug Administration, Building 50, Room 630, HFT 100, 3900 NCTR Road, Jefferson, AR 72079 USA; Drug Discovery and Development, London, UK; Divison of Systems Biology, National Center for Toxicological Research, Food and Drug Administration, Jefferson, AR 72079-9502 USA

**Keywords:** Kidney, Sex difference, Gene expression, Drug transporters, Age, Ethnicity, ATP-binding cassettes (ABCs), Solute carriers (SLCs)

## Abstract

**Background:**

Differences in expression of drug transporters in human kidney contribute to changes in pharmacokinetics and toxicokinetics of a variety of drug compounds. The basal expression levels of genes involved in drug transport processes in the kidney introduces differences in bioavailability, distribution, and clearance of drugs, possibly influencing drug efficacy and adverse reactions. Sex differences in gene expression of transporters are a key cause of differences in sex-dependent pharmacokinetics, which may characterize many drugs and contribute to individual differences in drug efficacy and toxicity. Therefore, evaluating the expression of drug transporters in normal human kidneys is important to better understand differences in drug bioavailability, distribution, and clearance of drugs in humans. Other factors such as age and ethnicity may also contribute to individual differences in gene expression of drug transporters in the human kidney.

**Methods:**

Quantitative real-time PCR (QRT-PCR) was performed to determine the gene expression of 30 drug transporters in 95 age-matched normal human kidney tissues. Multiple Student’s *t*-tests (Sidak-Bonferroni correction) and two-way ANOVA (Bonferroni correction) analyses were used to determine statistically significant differences.

**Results:**

In the 30 transporter genes examined, sex, ethnicity, and age differences in gene expression were exhibited in normal human kidney tissue. These changes in expression were not found to be differentially significant. However, sex-age and sex-ethnicity interactions were found to be statistically significant. For sex-age interactions, SCL22A12 was found to be significantly higher expressed in females <50 years compared to males <50 years. Expression levels of SLC22A2, SLC22A12, SLC6A16, and ABCB6 were significantly higher in females <50 years compared to females ≥50 years. In sex-ethnicity interactions, expression levels of ATP7B and KCNJ8 were found to be significantly higher in African American females compared to European American females. Also, the expression of SLC31A2 was significantly higher in European American males compared to European American females.

**Conclusions:**

Sex, age, and ethnic differences impacted the expression of drug transporters in normal human kidneys, which suggests that the analysis of gene expression of drug transporters will aid in improving the usage/dosage of drug therapies influencing personalized medicine and susceptibility to adverse drug reactions.

## Background

One of the main pharmacokinetic organs, the kidney, is responsible for maintaining fundamental physiological processes in the human body, such as the regulation of electrolytes, fluid balance, blood pressure, the production of various hormones, the reabsorption of key nutrients and other small molecules, and the metabolism, excretion, and transport of small molecules, drugs, and xenobiotics [1-3]. In the kidney, transporters, which are members of the ATP-binding cassette (ABC) and solute carrier (SLC) superfamilies, are responsible for recognizing and transporting metabolites, hormones, and organic substrates, such as pharmaceutical agents [4]. Drug transporters, in particular, ABCs (efflux transporters) and SLCs (uptake transporters), have been identified as accepted elements in the pharmacokinetics and toxicokinetics of a variety of drugs and have significant influence on a drug’s disposition, efficacy, and toxicity [5-7]. The variability of drug responses among individuals occurs because of complex and multifactorial contributions from genetic, environmental, disease/health, and drug-drug interactions [8]. These factors alter drug absorption, metabolism, and pharmacokinetics in humans and lead to inter-individual variability of drug efficacy, safety, and adverse drug reactions [6,8]. Therefore, understanding the underlying factors that affect the pharmacokinetics and pharmacodynamics of pharmaceutical agents in a diverse population is essential in improving therapeutic efficiency while diminishing adverse events. Many factors influence the concentration of a drug in circulation or at the site of action and determine the resulting outcomes; one of these factors is the sex of the individual [9-11].

Sex differences in drug efficacy and drug safety have been widely studied in many epidemiology studies as well in clinical investigations [1,10,12]. Based on these studies, sex differences in the expression of drug metabolizing enzymes and transporters (DMETs) are thought to be one of the most important determinants accounting for individual differences in clinical pharmacology, pharmacokinetics, and toxicokinetics leading to differences in drug efficacy and safety [1,7,12,13]. However, the majority of these studies focused on DMET processes occurring in the liver, another key pharmacokinetic organ, instead of the kidney. Therefore, the elucidation of sexually differentiated regulation of DMETs in healthy/normal human kidneys and understanding of its potential impact on adverse drug reactions is not fully understood.

Two other factors that influence pharmacokinetics and pharmacodynamics of drug transporters are age and ethnicity. In aging rats, the kidney is more susceptible to acute injury resulting in ischemic injury or drug-induced toxicity. Specific drugs have also been shown to require age-specific dosing [14-17]. Thus, understanding the relative influence of age on the renal expression of DMETs that influence the drug clearance and bioavailability is critical. Ethnicity is also thought to be a factor that may contribute to observed differences in both pharmacokinetics and pharmacodynamics of drugs, which can result in variability in drug therapy responses. Several studies have indicated that ethnic differences in DMETs that contribute to differences in drug response may be a consequence of diet, medical practices, or genetic differences [18-20]. Also, studies have shown that certain genetic differences in drug transporters that are associated with different ethnic groups lead to reduced function, whereas subgroups of the same ethnic group exhibited similarities in their genetic profiles [20]. However, differences in mRNA expression of drug transporters have not been extensively studied among different ethnic groups.

This study was designed to evaluate the inter-individual variability of renal drug transporters in normal human kidney tissue. Here, we investigated the mRNA expression of drug transporters, which were previously identified in a hepatic study [7], to determine whether or not these transporters were also expressed in the human kidney. The selected transporters were mainly comprised of the two main transporter categories: ABC and SLC families. Although sex and age differences have been previously reported in the kidney, most of the published data are from rat and mouse tissue or human cell lines, not normal human kidney tissue, which is a more appropriate model for characterizing sex, race, and age differences in humans [1,4,17,21]. This study was also motivated by the observation that data obtained from rats, the most commonly utilized model in preclinical drug discovery pharmacokinetics studies, exhibit numerous examples of discrepancies when compared to data obtained from humans [4,21]. Our results demonstrate that all but three of the drug transporters previously identified in liver are also expressed in the kidney. Further evaluation also showed that several of these transporters exhibited not only sex but age and/or ethnic differences in expression.

## Methods

### Human kidney tissue

Human kidney samples were purchased from the US Cooperative Human Tissue Networks (CHTN). Information about the tissue samples regarding ethnicity, sex, age, and diagnosis was provided. Only normal tissues confirmed by pathological analysis were utilized. Kidney tissues from 34 females and 61 male age-matched individuals were included in this study. Age and ethnicity information of the tissue sample are provided in Table 1. This project was reviewed by the National Center for Toxicological Research (NCTR) and Food and Drug Administration (FDA) Research Involving Human Subject Committee (RIHSC) and received an exempt status.

**Table 1:** **Subgrouping of 61 male and 34 female normal kidney tissues**

**Sex**	**Number**	**Ethnicity**	**Number**	**Age**	**Number**
Male	61			<50	2
		African American	11	≥50	9
				<50	10
		European American	48	≥50	38
				<50	2
		Others	2	≥50	0
Female	34			<50	2
		African American	11	≥50	9
				<50	4
		European American	21	≥50	17
				<50	1
		Others	2	≥50	1

### Isolation of RNA and complementary DNA (cDNA)

Frozen human kidney tissues were ground with a chilled mortar and pestle for quality RNA isolation. Total RNA was prepared from the samples using RNeasy Mini Kit (Qiagen, Valencia, CA, USA) according to the manufacturer’s protocol. cDNA was synthesized (reverse transcribed polymerase chain reaction (RT-PCR)) from 0.4 μg of high quality RNA (RIN > 8) using the Advantage RT-for-PCR Kit according to the manufacturer’s protocol (Clontech Laboratories Inc., Mountain View, CA, USA). The cDNA concentration was normalized to 20 ng/μl. Fifty nanograms of cDNA was used per sample reaction.

### Quantitative real time- polymerase chain reaction (QRT-PCR)

For QRT-PCR, SYBR master mix solution was obtained from Applied Biosystems (Carlsbad, CA, USA) and primers for GAPDH, ABCA5, ABCB1, ABCB6, ACCN4, ATP2B2, ATP7B, KCNJ8, KCNK5, MRP1, MXR, MRP5, OST-Beta, SLC10A1, SLC16A11, SLC22A12, SLC22A2, SLC22A9, SLC25A13, SLC30A1, SLC31A1, SLC31A2, SLC35F5, SLC4A1AP, SLC43A1, SLC5A6, SLC5A10, SLC6A16, SLC9A1, SLCO1B1, and TRPC4AP were obtained from Sigma-Aldrich (St. Louis, MO, USA). All samples were assayed in triplicate using a Biorad CFX96 C1000 system (Biorad, Hercules, CA, USA). ΔΔ*CT* calculations were used to determine fold change. GAPDH functioned as the reference gene. QRT-PCR was performed using the primers listed in Table 2. Expression change ratios greater than 1.5 were considered differentially expressed.

**Table 2:** **List of primers utilized in this study**

**Gene**	**Forward primer**	**Reverse primer**
GAPDH	5′-GAAGATGGTGATGGGATTTC-3′	5′-GAAGGTGAAGGTCGGAGTC-3′
MRP5	5′-TGAAAGCCATTCGAGGAGTTG-3′	5′-CGGAAAAGCTCGTCATGCA-3′
SLC5A10	5′-GATGTGCCCTTGGGAACTAAA-3′	5′-GGCATTGAAGCCACAGACA-3′
ABCB1	5′-AGTGTTGGACAGGCATC-3′	5′-CTCTGCACCTTCAGGTT-3′
ACCN4	5′-ACCGCTTCCGGCATTCGGC-3′	5′-CTGGGCAGTGAACTCTGAGG-3′
MXR	5′-CAGGTCTGTTGGTCAATCTCACA-3′	5′-TCCATATCGTGGAATGCTGAAG-3′
MRP1	5′-GAAGGCATCGGACTCTTCA-3′	5′-CAGCGCGGACACATGGT-3′
ATP2B2	5′-TTGGAGTAAAAGTCGCTGTTG-3′	5′-ATCACCCGGCAGCCTCTT-3′
ATP7B	5′-ATGAGAGCACCACAGACACAG-3′	5′-TGATTTATAACCTGGTTGGGAT-3′
KCNK5	5′-GTCTCCCCACCCCCGCTTGTC-3′	5′-CCCGCCGGTCGCCTCTTCTG-3′
KCNJ8	5′-GTGAGCCTGAGCTGTTTTCA-3′	5′-CATCTTTACCATGTCCTTCC-3′
SLC01B1	5′-TCTCTATGAGATGTCACTGGAT-3′	5′-TGAACACCGTTGGAATTGC-3′
SLC22A2	5′-CTGAGCTGTACCCCACATTCA-3′	5′-CAAGTACGCCGAAAACCATCA-3′
SLC22A12	5′-TGGTGCTAACCTGGAGCTACC-3′	5′-TGTTCATCATGACGCCTGC-3′
SLC22A9	5′-AGAAATGCAGACGCTGCGT-3′	5′-ACCAAGGGTATTGCAAGAGC-3′
SLC10A1	5′-ATGTTTGCCATGACACCACTC-3′	5′-CATAACCCAGCAGAAAGCCA-3′
SLC31A1	5′-TAAGATTCGGAGAGAGAGGTGC-3′	5′-AGGCTCTCTCGGGCTATCTT-3′
SLC6A16	5′-AAGACCAGAAGACAGACGGAGGA-3′	5′-CGAGGGCCCAAAGTTTGC-3′
SLC5A6	5′-TCCCTCTAATGGGTCCAGCTT-3′	5′-GACAAGGAATAGAACCGCTGC-3′
ABCB5	5′-GGCTGCTATTCTGACCACTCACTATA-3′	5′-TTAACTGCCCAGACACCATGAT-3′
OST-beta	5′-ATGGTCCTCCTGGGAAGAAGCA-3′	5′-GCCTCATCCAAATGCAGGACTTC-3′
SLC16A11	5′-CGTCGGAGGTGTGGTGCAGG-3′	5′-AGGAAAGAGGCGGTGAAGTCTC-3′
SLC25A13	5′-AGATGGTTCGGTCCCACTTGCA-3′	5′-ACCAGTGGTGATTTCTCCTGCC-3′
SLC30A1	5′-GGAGGAGACCAACACCCTGGT-3′	5′-GGAGGAGACCAACACCCTGGT-3′
SLC31A2	5′-ATCAGCCAGCAGACCATCGCAG-3′	5′-TGAAGTAGCCGATGACCACCTG-3′
SLC35F5	5′-GTGAACCTCTGTATGTGCCTGTG-3′	5′-TGACGGAAGCTGTCGAATCTCC-3′
SLC43A1	5′-GATGCTGGAGTACCTTGTGACTG-3′	5′-CAGGTGAGAAGGCACAACAGCT-3′
SLC4A1AP	5′-TGGTGCCATGAAAGGAGGAAGC-3′	5′-CCTCCTCTTCTACTTCAGGCTC-3′
SLC9A1	5′-GAACTGGACCTTCGTCATCAGC-3′	5′-GGTCAGCTTCACGATACGGAAC-3′
TRPC4AP	5′-AAGAAGGAGCCAGCAGAGTCGT-3′	5′-CCTCGCTTCAGCAGGAACATCT-3′

### Statistical analysis

All statistical analyses were performed using GraphPad Prism software version 6.0 (San Diego, CA, USA). The non-parametric Mann–Whitney was used to compare GAPDH expression in the normal human kidney tissue based on sex, age, or ethnicity. And non-parametric Kruskal-Wallis was used to compare GAPDH expression in the normal human kidney tissue based on sex-age and sex-ethnicity interactions. Multiple Student’s *t*-test was performed with the Sidak-Bonferroni correction method (alpha = 5%) to determine statistically significant differences in each group; sex, age, or ethnicity. Using multiple Student’s *t*-test with Sidak-Bonferroni correction, *p* < 0.05 was not considered significant. Analysis was significant when *p* < 0.0017. Two-way ANOVA with Bonferroni correction was used to determine statistically significant differences based on group interaction: sex and age or sex and ethnicity. Using two-way ANOVA with Bonferroni correction, *p* < 0.05 was determined to be significant. For power analysis, GraphPad Statmate 2 software (San Diego, CA, USA) was used to determine if the study was suitably powered to detect statistical difference.

## Results

**Figure 1:**
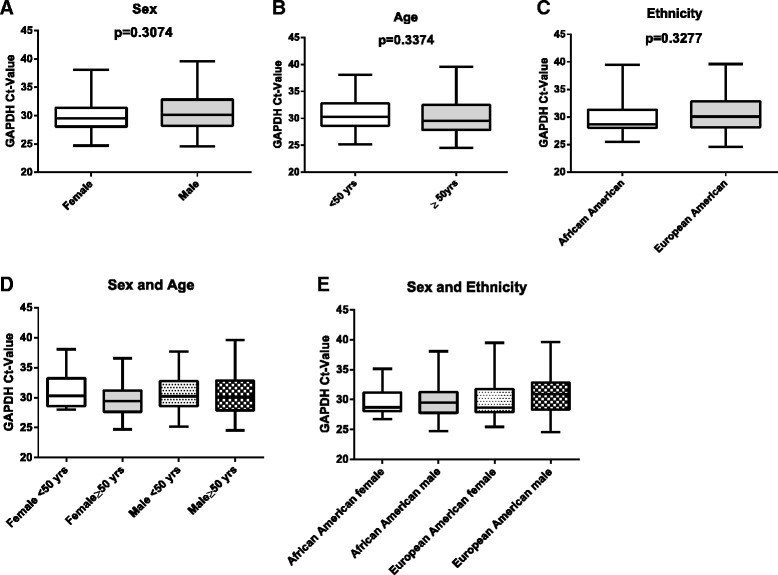
**Comparison of expression level for housekeeping gene, GAPDH, based on sex, age, and ethnicity.** Box-whisker plot demonstrates cycle threshold (Ct) values from lower to upper quartiles intersected by median for **(A)** sex, **(B)** age, **(C)** ethnicity**, (D)** sex-age interaction, and **(E)** sex-ethnicity interaction. Statistical analysis for sex, age, and ethnicity was performed using non-parametric Mann–Whitney test. Statistical analysis for sex-age and sex-ethnicity interaction was performed using non-parametric Kruskal-Wallis test (see Table 3).

The drug transporter genes selected for this study were detected in a previous Illumina Human Whole-Genome BeadChips (Human-6-v1; Illumina, San Diego, CA, USA) microarray-filtered analysis of age-matched human liver specimens that identified 1,640 genes differentially expressed according to sex [7]. Twenty-six of these genes were utilized to determine which genes were also differentially expressed in human kidney tissues. Four additional transporter genes, which were previously noted as being differentially expressed in the liver, MXR, MRP5, MRP1, and ABCB1, were also examined [22]. As part of mRNA expression analyses, an appropriate choice of housekeeping gene(s) is critical. To confirm the utilization of a single housekeeping gene, GAPDH, we averaged the GAPDH cycle threshold (Ct) value of the 95 human kidney samples as well as compared the average GAPDH Ct values based on sex, age, and ethnicity. The average the GAPDH Ct value (mean ± SEM) of the 95 human kidney samples was 30.47 ± 0.36. As shown in Figure 1, there was no significant difference in average GAPDH Ct values (mean ± SEM) based on sex (female: 29.94 ± 0.53 and male: 30.77 ± 0.48; Figure 1A), age (age < 50 years: 31.00 ± 0.71 and age ≥ 50 years: 30.32 ± 0.42; Figure 1B), and ethnicity (African American: 29.91 ± 0.65 and European American: 30.65 ± 0.45; Figure 1C). Further analysis showed no significant difference of GAPDH Ct values based on sex-age (Figure 1D, Table 3) and sex-ethnicity (Figure 1E, Table 3) interactions. Based on these findings, the use of a single reference gene, GAPDH, is acceptable and the observed change in regulation is reflective of the target gene not the reference gene.

**Table 3:** **Statistical evaluation of GAPDH levels based on sex-age and sex-ethnicity interactions**

**Sex-age**		**Sex-ethnicity**	
	**Krustal-Wallis** ***p*** **value**		**Krustal-Wallis** ***p*** **value**
Female <50 yrs vs. female ≥50 yrs	>0.9999	AA female vs. AA male	0.9998
Male <50 yrs vs. male ≥50 yrs	>0.9999	EA female vs. EA male	0.8630
Female <50 yrs vs. male <50 yrs	>0.9999	AA female vs. EA female	0.9967
Female ≥50 yrs vs. male ≥50 yrs	>0.9999	AA male vs EA male	0.6098

Statistical differences between GAPDH Ct for sex-age and sex-ethnicity interactions were assessed by non-parametric paired Krustal-Wallis test. *p* > 0.05 indicates suitability as housekeeping gene. AA, African American; EA, European American.

Of the 30 genes studied using GAPDH as the reference gene, three genes, SLCO1B1, SLC30A1, and ACCN4, whose mRNA expressions were detected in the liver, were not detected in the kidney. Studies were conducted on sex, ethnic, and age differences among the remaining 27 genes.

### Sex differences in the expression of transporters in human renal samples

**Figure 2:**
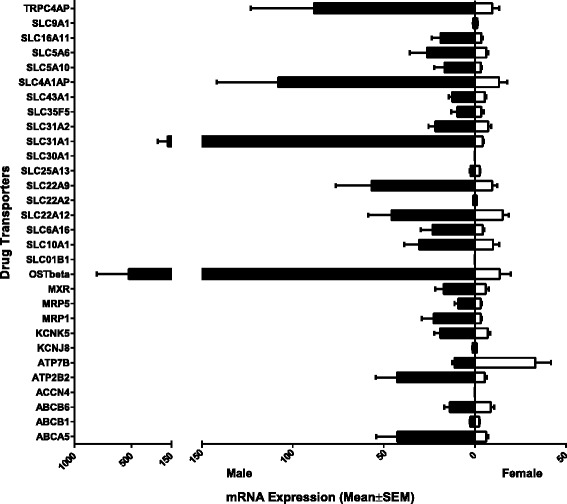
**Relative mRNA expression levels of drug transporters exhibiting sex differences in human kidney tissue.** The 30 transporter mRNA expressions for human kidney tissue were graphed to illustrated sex (male and female) differences. The bars represent the mean relative mRNA expression; the error bars indicate the standard error mean from *n* > 3 samples. Statistical analysis was performed using multiple Student’s *t*-test (Sidak-Bonferroni correction method (alpha = 5%)).

Filtering for differential expression by sex (fold change ratio >1.5), twenty-one transporter genes (ABCA5, ABCB6, MRP1, MRP5, MXR, OST-beta, KCNK5, ATP2B2, TRPC4AP, SLC10A1, SLC6A16, SLC22A12, SLC22A9, SLC31A1, SLC31A2, SLC35F5, SLC43A1, SLC4A1AP, SLC5A10, SLC5A6, and SLC16A11) exhibited higher expression in males compared to females (Figure 2). Two transporter genes (ATP7B and SLC9A1) exhibited higher expression in females compared to males, and the remaining four genes (ABCB1, KCNJ8, SLC22A2, and SLC25A13) were similarly expressed in males and females (Figure 2). However, when statistical analyses were performed using multiple Student *t*-test (Sidak-Bonferroni correction method), expression levels between male and female were not found to be significantly differentiated.

### Age differences in the expression of transporters in human renal samples

**Figure 3:**
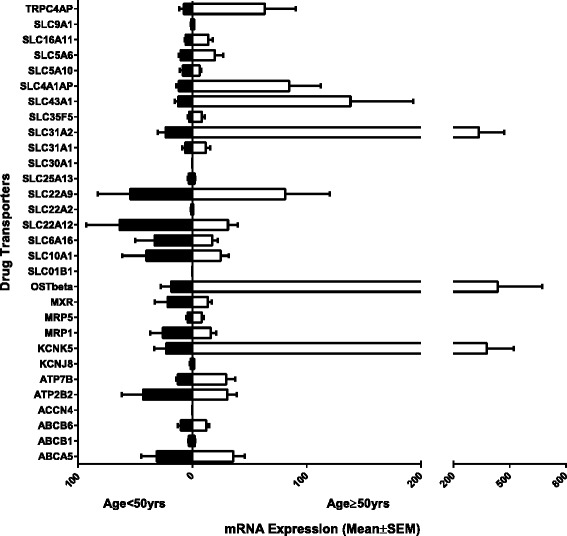
**Relative mRNA expression levels of drug transporters exhibiting age differences in human kidney tissue.** The 30 transporter mRNA expressions for human kidney tissue were graphed to illustrated age (age group: below 50 years old (<50 years) and age group: 50 years and older (≥50 years)) differences. The bars represent the mean relative mRNA expression; the error bars indicate the standard error mean from *n* > 3 samples. Statistical analysis was performed using multiple Student’s *t*-test (Sidak-Bonferroni correction method (alpha = 5%)).

The expression of the 27 drug transporters were further analyzed to identify genes that demonstrated age differences in gene expression in normal kidney tissue (fold change ratio >1.5). We divided the human kidney samples into two groups based on age: patients below 50 years old (<50 years) and patients 50 years and older (≥50 years). Here, we observed 13 transporter genes (MRP5, OST-Beta, KCNK5, ATP7B, TRPC4AP, SLC22A9, SLC31A1, SLC31A2, SLC35F5, SLC43A1, SLC4A1AP, SLC5A6, and SLC16A11) exhibiting higher mRNA expression levels in age ≥ 50 years compared to age <50 years (Figure 3). Seven transporter genes (MRP1, MRP5, SLC10A1, SLC6A16, SLC22A2, SLC22A12, and SLC25A13) exhibiting higher mRNA expression levels in age <50 years compared to age ≥ 50 years (Figure 3). And the remaining seven genes (ABCA5, ABCB1, ABCB6, ATP2B2, KCNJ8, SLC5A10, and SLC9A1) were similarly expressed in both age groups (Figure 3). This finding suggests age may also be a factor that contributes to differences in adverse responses in human kidney. Statistical analyses using multiple Student’s *t*-test (Sidak-Bonferroni correction method) between age <50 years and age ≥ 50 years were not found to be significantly differentiated.

### Sex and age differences in the expression of transporters in human renal samples

**Figure 4:**
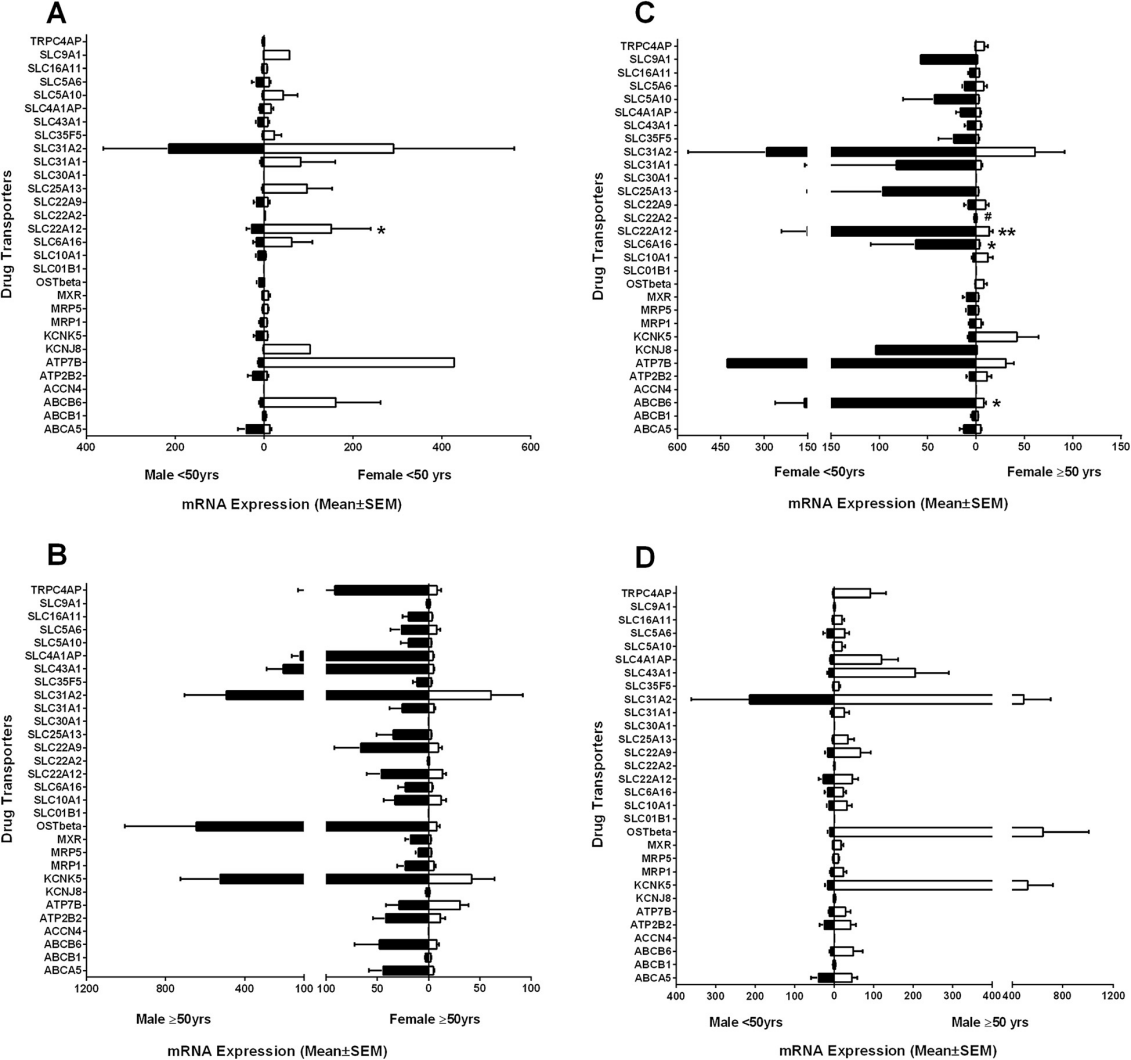
**Relative mRNA expression profiles exhibiting sex and age differences of drug transporters.** The 30 transporter mRNA expressions for human kidney tissue were graphed to illustrated sex and age differences: **(A)** males <50 years, **(B)** males ≥50 years, **(C)** females <50 years, and **(D)** females ≥50 years. The bars represent the mean relative mRNA expression; the error bars indicate the standard error mean from *n* > 3 samples. Statistical analysis was performed using two-way ANOVA (Bonferroni correction); *p < 0.05, **p < 0.05, and ^**#**^ *p* < 0.01.

Interestingly, when the two sexes were further analyzed and sub-grouped by age (females <50 years, males <50 years, females ≥50 years and males ≥50 years), we observed sex- and age-related differentiation in gene expression of drug transporters (Figure 4).

Evaluation of the expression levels of the 27 drug transporter genes in females <50 years and males <50 years identified 14 transporter genes (ABCB6, MRP5, MXR, KCNJ8, ATP7B, SLC6A16, SLC22A2, SLC22A12, SLC25A13, SLC31A1, SLC35F5, SLC4A1AP, SLC5A10, and SLC9A1) that were expressed higher in females <50 years compared to males <50 years. Using two-way ANOVA with Bonferroni correction, the difference in expression was significant for one of the transporter genes, SLC22A12 (*p* < 0.05). On the other hand, nine transporter genes (ABCA5, SLC5A6, SLC10A1, SLC22A9, SLC43A1, ATP2B2, KCNK5, OST-beta, and TRPC4AP) were expressed higher in males <50 years compared to females <50 years. And four transporter genes (ABCB1, MRP1, SLC16A11, and SLC31A2) were similarly expressed in males <50 years and females <50 years (Figure 4A).

Comparing the expression levels of the 27 drug transporter genes in females ≥50 years and males ≥50 years, we observed 24 transporter genes (ABCA5, ABCB6, MRP1, MRP5, MXR, OST-Beta, KCNK5, KCNJ8, ATP2B2, TRPC4AP, SLC10A1, SLC6A16, SLC22A12, SLC22A9, SLC25A13, SLC31A1, SLC31A2, SLC35F5, SLC43A1, SLC4A1AP, SLC5A10, SLC5A6, SLC16A11, and SLC9A1) that were expressed higher in males ≥50 years compared to females ≥50 years (Figure 4B). The difference in expression between females ≥50 years and males ≥50 years was not significant using two-way ANOVA (Bonferroni correction) analysis. And the remaining three transporter genes (SLC22A2, ATP7B, and ABCB1) were similarly expressed in females ≥50 years and males ≥50 years (Figure 4B).

When the expression levels of the 27 drug transporter genes were further investigated in females <50 years and females ≥50 years, we observed 20 transporter genes (ABCA5, ABCB1, ABCB6, MRP5, MXR, KCNJ8, ATP7B, SLC6A16, SLC22A2, SLC22A12, SLC25A13, SLC31A1, SLC31A2, SLC35F5, SLC43A1, SLC4A1AP, SLC5A10, SLC5A6, SLC16A11, and SLC9A1) that were expressed higher in females <50 years compared to females ≥50 years (Figure 4C). Based on two-way ANOVA with Bonferroni correction, females <50 years exhibited significantly higher mRNA expression levels of four of these drug transport genes; SLC22A2 (*p* < 0.01), SLC22A12 (*p* < 0.005), SLC6A16 (*p* < 0.05), and ABCB6 (*p* < 0.05) compared to females ≥50 years (Figure 4C). Five transporter genes (KCNK5, OST-beta, ATP2B2, SLC10A1, and TRPC4AP) were expressed higher in females ≥50 years compared to females <50 years (Figure 4C). And two transporter genes (SLC22A9 and MRP1) were similarly expressed in females ≥50 years and females <50 years (Figure 4C).

Comparing the expression levels of the 27 drug transporter genes in males <50 years and males ≥50 years, we observed 23 transporter genes (ABCB6, MRP1, MRP5, MXR, OST-Beta, KCNK5, KCNJ8, ATP7B, ATP2B2, TRPC4AP, SLC10A1, SLC22A12, SLC22A9, SLC25A13, SLC31A1, SLC31A2, SLC35F5, SLC43A1, SLC4A1AP, SLC5A10, SLC5A6, SLC16A11, and SLC9A1) that were expressed higher in males ≥50 years compared to males <50 years (Figure 4D). And the remaining four transporter genes (ABCA5, ABCB1, SLC6A16, and SLC22A2) were similarly expressed in males <50 years and males ≥50 years (Figure 4D). None of the genes were significantly differentiated in expression between males ≥50 years and males ≥50 years.

These findings suggest that the differences in drug transporter mRNA expression levels may be influenced by more than one factor. Here, we show the age of the different sexes (male and female) can influence the mRNA expression levels of several drug transporters.

### Ethnic differences in the expression of transporters in human renal samples

**Figure 5:**
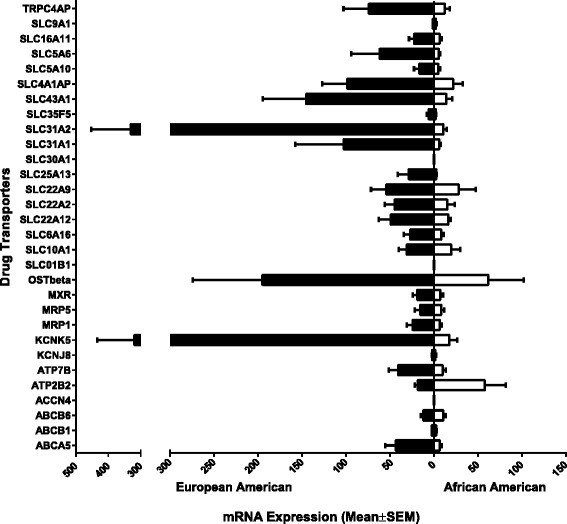
**Relative mRNA expression levels of drug transporters exhibiting ethnicity differences in human kidney tissue.** The 30 transporter mRNA expressions for human kidney tissue were graphed to illustrated ethnic (European American and African American) differences. The bars represent the mean relative mRNA expression; the error bars indicate the standard error mean from *n* > 3 samples. Statistical analysis was performed using multiple Student’s *t*-test (Sidak-Bonferroni correction method (alpha = 5%)).

Differential expression by ethnic groups, European American and African American, was also evaluated. Twenty-two transporter genes (ABCA5, MRP1, MRP5, MXR, OST-Beta, KCNK5, ATP7B, TRPC4AP, SLC10A1, SLC6A16, SLC22A2, SLC22A12, SLC22A9, SLC25A13, SLC31A1, SLC31A2, SLC35F5, SLC43A1, SLC4A1AP, SLC5A10, SLC5A6, and SLC16A11) exhibited higher expression in European Americans compared to African Americans (Figure 5). In contrast, two transporter genes (ATP2B2 and SLC9A1) exhibited higher expression in African Americans compared to European Americans (Figure 5). And three transporter genes (ABCB1, ABCB6, and KCNJ8) were similarly expressed in European Americans and African Americans (Figure 5). This finding suggests ethnicity may also be a factor that contributes to differences in adverse responses in human kidney. Using multiple Student’s *t*-test (Sidak-Bonferroni correction method), statistical analyses were performed between the expression levels of European American and African Americans were found not to be significantly differentiated.

### Sex and ethnic differences in the expression of transporters in human renal samples

Further analysis was performed to evaluate the gene expression differences of the two sexes sub-grouped by ethnicity (African American Males vs. European American Males or African American Females vs. European American Females).

**Figure 6:**
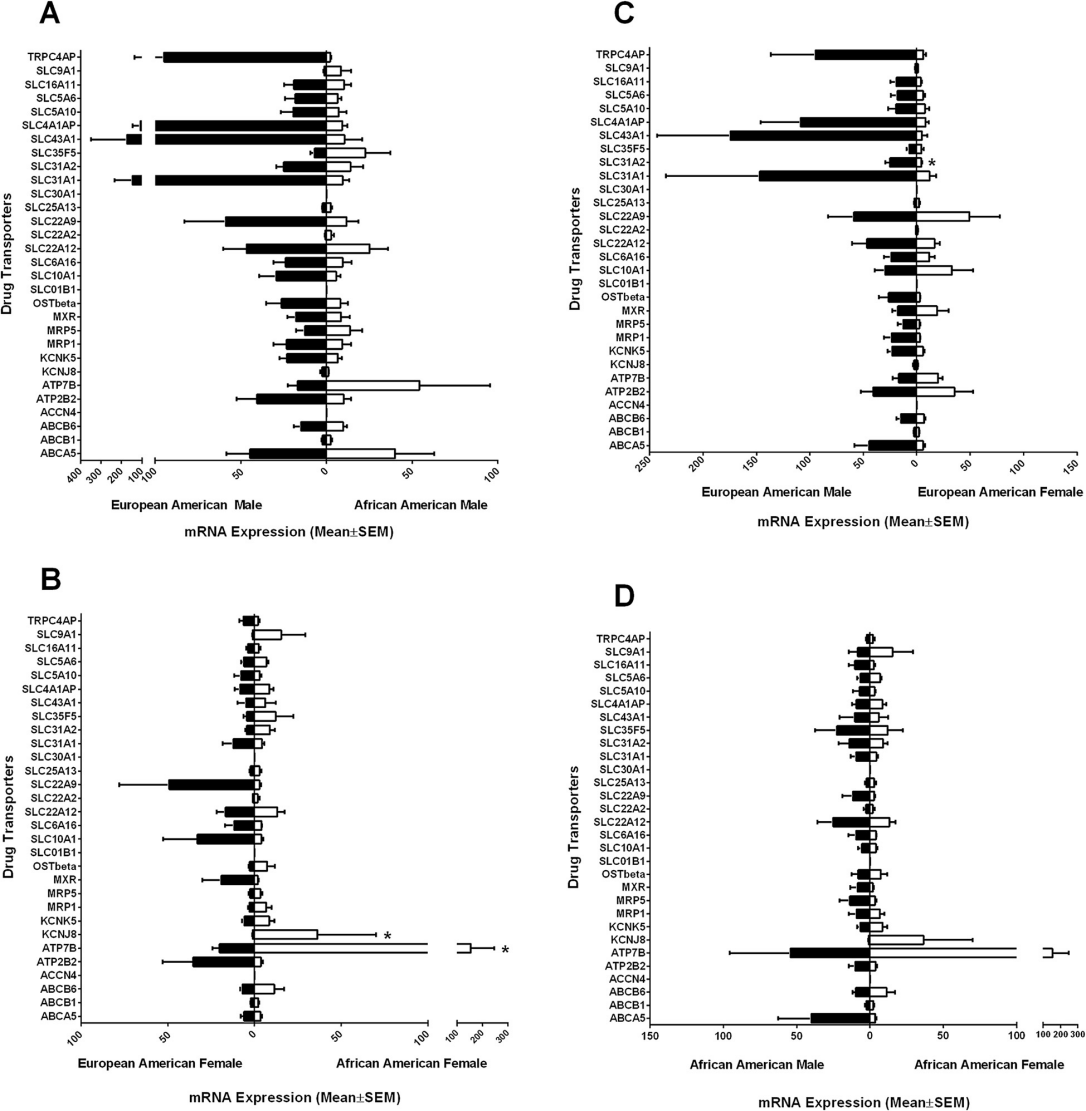
**Relative mRNA expression profiles exhibiting sex and ethnic differences of drug transporters.** The 30 transporter mRNA expressions for human kidney tissue were graphed to illustrated sex and ethnic differences: **(A)** European American males, **(B)** European American females, **(C)** African American males, and **(D)** African American females. The bars represent the mean relative mRNA expression; the error bars indicate the standard error mean from *n* > 3 samples. Statistical analysis was performed using two-way ANOVA (Bonferroni correction); *p < 0.05.

When the expression levels of the 27 drug transporter genes were further investigated in European American males and African American males, 19 transporter genes (ABCB6, MRP1, MXR, OST-Beta, KCNK5, KCNJ8, ATP2B2, TRPC4AP, SLC10A1, SLC6A16, SLC22A12, SLC22A9, SLC31A1, SLC31A2, SLC43A1, SLC4A1AP, SLC5A10, SLC5A6, and SLC16A11) exhibited higher expression in European American males compared to African American males (Figure 6A). On the other hand, four transporter genes (ATP7B, SLC22A2, SLC35F5, and SLC9A1) exhibited higher expression in African American males compared to European American males (Figure 6A). And four transporter genes (ABCA5, ABCB1, MRP5, and SLC25A13) were similarly expressed in European American and African American males (Figure 6A). None of the genes found to be differentiated were significantly expressed in European American males and African American males.

Comparing the expression levels of the 27 drug transporter genes in European American females and African American females, ten transporter genes (MXR, ATP2B2, TRPC4AP, SLC10A1, SLC6A16, SLC22A9, SLC31A1, SLC5A10, ABCA5, and SLC16A11) exhibited higher expression in European American females compared to African American females. Nine transporter genes (ABCB6, SLC31A2, MRP1, ATP7B, SLC22A2, OST-beta, KCNJ8, SLC35F5, and SLC9A1) the differences in expression level were significant (Figure 6B). Our finding showed that for two of these genes, ATP7B (*p* < 0.0.5) and KCNJ8 (*p* < 0.05), which exhibited higher expression in African American females compared to European American females were found to be significant differences in mRNA expression in the human kidney based on two-way ANOVA with Bonferroni correction. The remaining eight genes (KCNK5, ABCB1, MRP5, SLC22A12, SLC43A1, SLC25A13, SLC4A1AP, and SLC5A6) were similarly expressed in European American and African American females (Figure 6B).

When the two ethnic groups, European American and African American, were sub-grouped by sex, and gene expression differences were evaluated between European American females and European American males, 18 transporter genes (ABCA5, ABCB6, MRP1, MRP5, OST-Beta, KCNK5, KCNJ8, TRPC4AP, SLC6A16, SLC22A12, SLC31A1, SLC31A2, SLC35F5, SLC43A1, SLC4A1AP, SLC5A10, SLC5A6, and SLC16A11) were found to be higher expressed in European American males compared to European American females (Figure 6C). Based on two-way ANOVA with Bonferroni correction, our finding demonstrated that one gene, SLC31A2 (*p* < 0.05), exhibited significant differences in mRNA expression (Figure 6C). The remaining nine genes (ABCB1, ATP2B2, ATP7B, MXR, SLC10A1, SLC22A9, SLC22A2, SLC25A13, and SLC9A1) were similarly expressed in European American females and males (Figure 6C).

The expression levels of the 27 drug transporter genes were further evaluated in African American males and African American females. Fourteen transporter genes (ABCA5, MRP5, MXR, ATP2B2, SLC6A16, SLC22A2, SLC22A12, SLC22A9, SLC31A1, SLC31A2, SLC35F5, SLC43A1, SLC5A10, and SLC16A11) exhibited higher expression in African American males compared to African American females (Figure 6D). Three transporter genes (ATP7B, KCNJ8, and SLC9A1) exhibited higher expression in African American females compared to African American males (Figure 6D). And the remaining ten genes (ABCB1, ABCB6, KCNK5, MRP1, OST-beta, SLC10A1, SLC25A13, SLC4A1AP, SLC5A6, and TRPC4AP) were similarly expressed in African American males and females (Figure 6D). None of the genes were found to be significantly differentiated in expression in African American males and African American females.

The findings of the study strengthens our suggestion that the differences in drug transporter mRNA expression levels may also be influenced by more than one factor and all three factors: sex, age, and ethnicity, impact the mRNA expression levels of drug transporters in the kidney.

## Discussion

In the kidney, tissue expression levels of drug transporters can mediate the selective filtering, retention, and excretion of renal drug and their metabolites [1-4]. However, there are little data available about the expression levels of drug transporters in normal human kidney, and most of the existing data has been obtained from rat, mice, or cell culture models. For this reason, the data previously presented are sometimes conflicting. Studies have shown that the expression of drug transporters in human kidney cell lines and rat tissue do not always correlate with the expression in human tissue from kidney [4]. These studies have shown in human kidney tissue, for example, that the transporter SLC22A1 (OCT1) is more commonly expressed while in rat kidney tissue SLC22A2 (OCT2) and SLC22A3 (OCT3) are most commonly expressed [4]. Differences in mRNA expression of drug transporters have also been demonstrated between kidney cell lines and human kidney tissue [4]. For example, SLC22A2 (OCT2) mRNA expression was detected in human kidney tissue but not in the human kidney cell line Caki-1; instead these cells expressed SLC22A3 (OCT3) [4]. Therefore, the use of kidney cell lines and rat tissue as a model for understanding drug transporters in human kidney should be approached with caution. For this reason, we focused on the use of human kidney tissue to assess the mRNA expression of drug transporters in determining sex, ethnic, and age differences in human kidney tissues. Tissue derived from human kidney serves as a more appropriate model for assessing the expression of drug transporters in the human kidney.

In this study, we utilized mRNA expression profiles to investigate the expression pattern of the 30 transporter genes. Our results and conclusions are based on mRNA expression data, which may not always correlate with the expression of protein. Therefore, the mRNA expression data shown for each gene must be considered as a starting point for assessing sex, ethnic, and age differences in human kidney tissues. The assessment of differential gene expression of drug transporters as a function of sex, age, as well as ethnicity was the focus of this study.

One of the most critical aspects of mRNA expression analyses is finding an appropriate choice of housekeeping gene. Several studies have shown that using a single housekeeping gene as a reference for renal studies may reflect regulation of the reference gene rather than the target gene [23,24]. These studies have shown that reference genes have the potential of being differentially regulated based on cell or tissue type as well as under certain conditions such as cell proliferation and cancer [23-34]. In recent years, an increasing number of publications have indicated the need of multiple/combination of housekeeping genes be considered as reference for QRT-PCR. Several studies have been conducted to identify acceptable housekeeping genes for QRT-PCR analyses using human renal samples. Dupasquier et al. [23] evaluated eight potential housekeeping genes for their expression level and stability to minimize inter-individual variations between clear cell renal cell carcinoma (ccRCC) samples and their corresponding healthy tissue. Of the eight housekeeping genes studied, peptidylpropyl isomerase A (PPIA also called cyclophilin A) and ribosomal protein S13 (RPS13), especially in combination, were best suitable to normalize gene expression in cancer and normal tissue [23]. In another study, Schmid et al. [24] assessed three commonly used housekeeping genes, GAPDH, 18SrRNA, and cyclophilin A, in 165 human kidney biopsies, which consisted of 22 controls (normal tissue) and 143 nephropathy tissues with a variety of histopathologic diagnoses. The data from this study recommended the use of 18SrRNA and cyclophilin A as housekeeping genes for gene expression analysis of tubulointersitial compartments and non-microdissected kidney biopsies [24]. Both studies focus on differences in the expression of the housekeeping gene under two different conditions, normal and cancer/diseased tissue, and indicate that using two or more housekeeping genes allows for the detection of any systematic bias that may arise. Here, we are focused on only one condition, normal tissue, and used GAPDH as the reference gene. GAPDH remains the most widely used housekeeping gene employed in QRT-PCR gene expression analyses and, based on our study parameters, is an acceptable reference control. Our findings indicate GAPDH as a reference gene in our study does not significantly influence the observed changes in drug transporter mRNA expression.

We determined that genes were differentially expressed if their expression ratio was greater than 1.5. A difference in expression was observed between the sex, age, and ethnicity as shown in Figures 2, 3, and 5, respectively. However, based on statistical analysis performed, multiple Student’s *t*-test with Sidak-Bonferroni correction method (alpha = 5%), gene expression between males and females, while differentially expressed were not found to be significant. This trend was also observed with age-only (age: <50 years and age: ≥50 years) and ethnic-only (European American and African American) analyses. The lack of statistical significance in the study may be due to genomic variations displayed in human tissue samples, which modulate their expression as well as the sample size. To determine if the study was suitably statistically powered to detect differences, we evaluated the power of the completed study. Using the sample sizes and the standard deviation observed, it was concluded that sex-only, age-only, and the ethnicity-only analyses have an average of 53%, 22%, and 28% chance (power), respectively, to detect significant difference. Therefore, a larger sample size may increase the significance of this study.

Our results showed that three transporters, ACCN4, SLCO1B1, and SLC30A1, were not expressed in the human kidney tissues. SLCO1B1, a liver-specific member of the organic anion transporter (OAT) family, and ACCN4 have been found mainly in the liver [11,35,36]. SLC30A1 has been found to be expressed in both human embryonic kidney 293 cells and normal human kidney tissue. These findings need to be evaluated further.

The remaining 27 transporters previously found to be expressed in the liver were found to be expressed in the human kidney. Of these genes, 23 were found to exhibit sex-bias in their mRNA expression levels, with 21 of the transporter genes exhibiting male dominance in expression and two of the transporter genes exhibiting female dominance in expression. Awareness of sex differences in response to drug bioavailability, distribution, and clearance is clinically important. Sex differences have been shown to contribute to adverse drug responses, which has drawn significant attention in recent years as many studies have shown women having a greater risk of suffering adverse drug responses than men [1,7,8,10,12,13]. For example, aspirin, an anti-inflammatory agent, is more rapidly cleared from women and its metabolite, salicylate, has an increased rate of absorption in women [11,36]. On the other hand, agents such as gemcitabine, a chemotherapeutic drug, and heparin, an anticoagulant, have a lower clearance rate in women [11,23]. Sex-specific differences in expression levels of drug transporters have been implicated in the induction of sex-specific toxicities in the kidney contributing to sex-related renal disease and injuries [37-40]. For example, male rats lack the sex-specific protection against mercury-induced nephrotoxicity observed in female rats [38]. One of the drug transporters that exhibited male dominance bias in this study was MRP, which was seven times (7×) more highly expressed in males compared to females. MRP1, a member of ABC superfamily, has been identified as a transporter for the pharmaceutical agent, adefovir [35]. Initially, the use of adefovir was proposed for the treatment of HIV. This was not approved by the Food and Drug Administration due to concerns about the severe and frequent cases of kidney toxicity associated with high doses of the drug [41]. This drug was repurposed and approved for the treatment of chronic hepatitis B virus infections at lower doses [41]. The warning label for adefovir indicates the risk of adverse drug reactions is higher in women. Our finding is supportive as we demonstrated that males have higher mRNA expression levels of MRP1 compared to females. The sex-biased expression pattern of drug transporters may provide explanations for clinical observations of sex-dependent pharmacokinetics. However, focused studies are needed to determine whether sex differences affect drug transport, adverse drug interactions, or drug-drug interactions.

Aging is another factor that contributes to differences in pharmacokinetic and pharmacodynamic behaviors. Aging is associated with a decline in the ability to metabolize and transport some drugs but there are very little data available on the clinical outcomes of dose modification, particularly at ages greater than 50 years (>50 years). For most pharmaceutical agents, dosage adjustment in older people is restricted to pharmacokinetic studies in small populations of healthy volunteers [5]. In older people, age-related changes in the pharmacokinetics and pharmacodynamics of drugs may increase the risks of toxicity or limit efficacy [14-16,42]. In addition, elder patients may demonstrate variable degrees of membrane nephropathy and nephrosclerosis, which affect the kidney function [43]. Recent studies have identified novel mechanisms for age-related changes in hepatic pharmacology and toxicology [5,9,44]. Zhang et al. [44] showed in mice liver that aging has been associated with decreased mRNA expression of solute carrier superfamily, specifically the solute carrier organic anion (SLCO) subfamily and solute carrier subfamily 22 (SLC22s). Furthermore, the mRNA expressions of several efflux transporters, including multidrug resistant proteins (MRP-2, MRP6, and MRP3), have been shown to be significantly reduced in livers of older mice [44]. In rat tissue, Kwekel et al. [17] demonstrated that two drug transporters, SLCO1A1 and SLC22A7, exhibited different expression patterns during the aging cycle. SLCO1A1 mRNA expression at the early age of 2 weeks showed low expression levels followed by a rapid increase in expression levels from age 5 to 21 weeks, followed by diminishing expression levels into adulthood. On the other hand, SLC22A7 was expressed at low levels between ages 2 and 8 weeks with a significant increase in expression levels in later adulthood in rats [17]. However, what this means in terms of how it translates to humans remains undetermined. In this study, we identified drug transporter genes with age-related changes in mRNA expression in human kidney tissue. Here, we showed that 13 of the 27 drug transporter studied exhibited an age-bias with individuals aged 50 or greater (≥50) years showing dominance and seven of the 27 drug transporter studied exhibited an age-bias with individuals aged less than 50 (<50) years showing dominance in mRNA expression. Due to the fact that the majority of age-related studies were performed in rats, limited data in normal human tissue are available to compare with our data. Other studies have also highlighted sex and age-associated changes in drug pharmacokinetics in persons of older ages [6,16,45]. Here, we demonstrated that the expression level varies among the sub-groups of sex and age: males <50 years, males ≥50 years, females <50 years, and females ≥50 years. We showed that women of age less than 50 (<50) had significantly higher levels of mRNA expression for the four drug transporters, SLC22A12, SLC22A2, SCL6A16, and ABCB6. These age-dependent changes in drug transporter expression in women may be due to changes in the levels of hormones such as estrogen [46,47] as well as medication use. Studies have implicated higher use of medication in women [48] and elderly [16]. Also, several medications have been shown to potentially influence expression of drug transporters, e.g., steroids may upregulated P-gp [49].

Ethnic inequality in treatment outcomes is a problem that is receiving increased recognition in clinical oncology. It is not completely clear whether differences noted between European Americans and African Americans in treatment outcomes are due to matters of patient access to care, differences in medical care delivery, differences in clinical response to the same therapies, genetic variability or, most likely, a combination of these factors [50]. Man et al. [20] assessed the genetic variations of DMETs across three major ethnic populations: Africans, Caucasians, and East Asians. They demonstrated that genetic differences occurred among the three major ethnic groups, which accounted for observed differences in their respective drug transporter function while among the East Asian subgroups, Chinese, Korean, and Japanese, genetic similarities were observed [20]. In this study, we focused on differences in gene expression between African American and European Americans for drug transporters, which may aid in our understanding of racial disparity demonstrated in treatment outcomes. In this study, we observed 22 of the 27 transporter genes studied exhibiting higher expression in European American compared to African Americans. And two of the 27 transporter genes studied exhibiting higher expression in African American compared to European Americans. One of these genes, SLC22A2 (OAT2), which is expressed three times (3×) higher in European Americans compared to African Americans, has been identified as a transporter for cisplatin. Cisplatin, a platinum-based anticancer agent, has been shown to induce higher rates of nephrotoxicity in African Americans [19]. Our data showed lower expression levels of SLC22A2, an uptake drug transporter, in African Americans. These data imply that other drug transporters with the ability to transport cisplatin or epigenetic factors may be contributing to cisplatin-induced nephrotoxicity observed in African Americans. The differences in drug transporters gene expression between different ethnicities as well the influence of ethnicity on sex may contribute to clinical treatment outcomes.

## Conclusions

The kidney is one of the main pharmacokinetic organs; therefore, the ability to make regulatory decisions regarding the impact of drugs and other chemicals on the kidney is dependent upon understanding of kidney biology and, more specifically, the expression profiles of the molecular components that play a role in pharmacokinetics, pharmacodynamics, and toxicokinetics in kidney tissue. Most studies have utilized rat tissue as an *in vivo* model. Our findings on the expression of drug transporters in human kidney tissue and the identification of factors that influence the drug transporter expression, sex, age, and/or ethnicity, improve our understanding of the impact of active drug transport processes on the pharmacokinetics and distribution of drugs in human kidney. The sex, age, and ethnic differences of drug transporters in the normal human kidney may be due to epigenetic modifications of these genes, a factor that needed to be further considered. With larger sample sizes, the differences in expression levels observed may be found to be significant. Our data also suggest that the three factors: sex, age, and race, may, at times, simultaneously influence adverse drug responses in the human kidney and drug-drug interactions.

## Abbreviations

QRT-PCR: Quantitative real-time polymerase chain reaction
ABCs: ATP-binding cassettes
SLCs: Solute carriers
DMETs: Drug metabolizing enzymes and transporters
CHTN: Cooperative Human Tissue Networks
cDNA: Complimentary deoxyribonucleic acid
RT-PCR: Reverse transcribed polymerase chain reaction
FDA: Food and Drug Administration
RIHSC: Research Involving Human Subject Committee
AA: African American
EA: European American
ccRCC: Clear cell renal cell carcinoma
GAPDH: Glyceraldehyde-3-phosphate dehydrogenase
RNA: Ribonucleic acid
18S rRNA: 18S ribosomal ribonucleic acid
PPIA: Peptidylpropyl isomerase A or cyclophilin A
RPS13: Ribosomal protein S13
OAT: Organic anion transporter
MRP: Multidrug-resistant proteins
mRNA: Messenger ribonucleic acid
KCN: Potassium family subfamily
